# Landscape and Season Influence Bat Activity and Richness in a Mediterranean Metropolitan Area

**DOI:** 10.1002/ece3.71978

**Published:** 2025-08-13

**Authors:** Vincenzo Meola, Ioannis Ekklisiarchos, Luca Cistrone, Antonello Migliozzi, Danilo Russo

**Affiliations:** ^1^ Laboratory of Animal Ecology and Evolution (AnEcoEvo), Dipartimento di Agraria Università degli Studi di Napoli Federico II Portici Italy; ^2^ NBFC, National Biodiversity Future Center Palermo Italy; ^3^ Natural History Museum of Crete University of Crete Heraklion Greece; ^4^ Dipartimento di Agraria Università degli Studi di Napoli Federico II Portici Italy

**Keywords:** bat, city, green areas, Naples, park, seasonality, urbanization

## Abstract

Urbanisation alters landscapes and filters biodiversity, yet its effects in Mediterranean cities remain poorly understood despite their unique ecological and climatic context. As adaptable insectivores and key providers of ecosystem services, bats are an important component of urban biodiversity. We investigated how landscape composition, artificial illumination, and seasonality shape bat activity and species richness in Naples (Southern Italy), a densely inhabited Mediterranean metropolis. We hypothesized that: (1) artificial illumination would favor light‐tolerant species; (2) urban areas would enhance bat activity through roosting and foraging opportunities; and (3) urban warming would reduce seasonal declines in activity. Passive ultrasonic recorders were deployed in summer and winter across 12 1 × 1 km cells spanning a gradient of green space size and fragmentation. We recorded seven species and one Myotis group, with communities dominated by synurbic taxa ( *Pipistrellus kuhlii* , *Hypsugo savii* , *Pipistrellus pipistrellus* ). Artificial illumination did not influence activity, and no species responded positively to light. Urban land cover and Mediterranean shrublands reduced both total activity and species richness, and the hypothesis that urbanization would enhance foraging opportunities for tolerant species was rejected. In contrast, urban natural parks increased bat activity and richness, favoring *H. savii* and *Tadarida teniotis* , while *P. pipistrellus* preferred open rural areas but avoided intensively managed agricultural land.*Pipistrellus* Seasonality emerged as the most consistent driver: bat activity and richness declined markedly in winter for all species, including presumed urban exploiters such as *P. kuhlii* and *H. savii* . Even the mild Mediterranean climate and urban heat island effects did not eliminate strong seasonal patterns. Mediterranean cities act as environmental filters, supporting only a few tolerant species. Urban natural parks provide critical refugia and should be prioritized alongside habitat heterogeneity, reduced pesticide use, and light pollution mitigation. Despite urban warming, pronounced winter declines persist, highlighting the need for continued monitoring to detect climate‐driven phenological shifts.

## Graphical abstract

Urbanization strongly shapes bat communities, yet Mediterranean cities remain understudied. We investigated how landscape composition, artificial illumination, and seasonality affect bat activity and richness in Naples, Italy, using passive acoustic monitoring across a gradient of urban green space and fragmentation. Species richness and activity were dominated by synurbic taxa, declined sharply in winter, increased in urban natural parks, and were negatively influenced by urban areas. Our work highlights the critical role of urban green spaces for sustaining bat diversity.

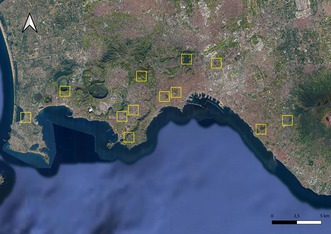

## 1 Introduction

Cities are expanding at a rate twice that of population growth, resulting in urban land now covering almost 0.5% of the Earth's surface, with continued rapid expansion projected (Angel et al. 2011). Despite occupying a small proportion of the Earth's surface, urban areas concentrate over half of the global population and up to 90% of economic activity (Schneider et al. 2010). This makes urban areas a critical arena for sustainability. Given their ecological footprint and potential, greater attention to urban biodiversity is essential, especially if we are to manage the ecosystem services it provides.

Urbanization, which covers low‐ to high‐density areas, is a significant and increasing factor in transforming landscapes and replacing natural habitats (van Vliet 2019). This process can largely impact biodiversity, favoring some species while negatively affecting others. Most species are excluded by the harsh conditions found in urban areas, but some can tolerate or even thrive in these environments (Santini et al. 2019; Alba et al. 2025). Wildlife responses to urbanization are often categorized into three main groups: urban avoiders, which are most common in natural or minimally disturbed habitats; urban adapters, capable of surviving in moderately urbanized areas; and urban exploiters, which reach their highest numbers in heavily urbanized settings (Blair 1996; McKinney 2002). Some researchers have also identified urban generalists (species that are relatively evenly distributed across the urbanization gradient) though this group is less often recognized as a distinct category (e.g., Francis and Chadwick 2012). Furthermore, some species display seasonal shifts in their use of urban areas, usually driven by changes in resource availability or environmental conditions in the surrounding natural habitats (Pais de Faria et al. 2021).

Since human population is constantly concentrating in urban areas and the trend keeps increasing, interactions between urban wildlife and urban citizens are exponentially growing (González‐Crespo et al. 2023). Urban biodiversity plays a crucial role in providing ecosystem services, including pest control, pollination, and seed dispersal, which enhance human well‐being and contribute to sustainable urban development (Ceauşu et al. 2019). However, ecosystem disservices may also arise from the presence of opportunistic or invasive animal species (Ceauşu et al. 2019). Therefore, a deep understanding of such interactions is pivotal in promoting ecosystem services delivered by wildlife and mitigating the consequences of ecosystem disservices.

Bats are among the most diverse groups of mammals, with 1498 species described worldwide (Simmons and Cirranello 2025), and they are found in nearly every terrestrial ecosystem, including urban areas (Russo and Ancillotto 2015; Santini et al. 2019). Many bat species make use of cities thanks to their ecological flexibility, roosting in artificial structures, foraging in green spaces or near streetlamps, and enduring despite significant human disturbance (Russo and Ancillotto 2015). In urban settings, bats provide vital ecosystem services such as insect pest control and, in some cases, pollination and seed dispersal (Russo et al. 2023). However, their presence in cities can pose challenges. Conflicts may occur when bats roost in buildings used by humans, raising concerns about property damage and public health (Voigt et al. 2016).

Understanding bat ecology in urban environments is essential for improving their ecosystem benefits and reducing potential disservices. Urbanization generally causes habitat loss, fragmentation, and degradation, all of which negatively impact bat populations (Russo and Ancillotto 2015). Although bats' ability to fly enables them to cross urban areas and access fragmented habitats, urbanization is often linked with decreases in bat diversity and numbers. Responses to urban development vary greatly among species, reflecting a broad spectrum of ecological traits and tolerances (Santini et al. 2019). Therefore, bats serve as a particularly informative group for evaluating the effects of urbanization. Their sensitivity to environmental changes and ecological functions makes them valuable bioindicators for tracking human‐induced landscape changes, including in urbanized areas (Russo et al. 2021).

Artificial Light at Night (ALAN) is a widespread feature of urban areas that can significantly influence bat behavior, especially foraging and commuting activities. While some species are tolerant of light and may benefit from insect congregations near streetlights, others completely avoid lit areas (Mathews et al. 2015; Russo et al. 2019). These differing responses make ALAN a crucial aspect to explore when studying urban bat ecology. Street lamp foraging by light‐opportunistic bat species results from a trade‐off between increased predation risk in lit conditions and prey availability (Rydell 1992). Insect‐poor habitats where lighting does not concentrate enough arthropod prey may be avoided, while those where light attracts abundant prey (and impairs antipredatory tympanate prey's responses) may cause greater bat activity (Mathews et al. 2015; Russo et al. 2019; Salinas‐Ramos et al. 2021).

Green spaces also play a crucial role in urban bat ecology by providing foraging opportunities, roosting sites, and commuting corridors. Parks, woodlands, and low‐intensity agricultural mosaics have been shown to support higher bat richness and activity, particularly for edge‐ and clutter‐adapted species (Tena et al. 2020; Callas et al. 2024). The amount and fragmentation of green areas may thus significantly shape urban bat assemblages.

Lastly, seasonality in bat activity, particularly in temperate and Mediterranean climates, is influenced by insect prey availability and temperature constraints. Although urban heat island effects may reduce temperature drops and possibly enable year‐round activity, winter declines are still generally observed even in mild climates (Fontanarrosa et al. 2009; Mas et al. 2022). Determining whether urban warming lessens seasonal effects in Mediterranean cities remains an open question.

Despite growing interest in urban bat ecology, Mediterranean urban systems remain underrepresented in the literature. Naples (Southern Italy) is one of the largest cities in Southern Europe. It offers a unique study system due to its densely built landscape interspersed with parks, agroecosystems, and heterogeneous green space. No previous study has systematically assessed how habitat features, ALAN, and seasonality influence urban bat communities in such a context. The mild Mediterranean climate and the heterogeneous mosaic of green spaces in Naples may modulate bat responses to urbanization, artificial illumination, and seasonality. This context warrants specific investigation to determine whether these factors interact differently compared with other temperate cities. Here, we aimed to fill this gap by assessing how landscape composition, ALAN, and season influence bat activity and richness in this metropolitan area. Based on prior evidence, we developed the following hypotheses:

1. ALAN would favor light‐tolerant species such as *P. kuhlii* and *H. savii* , both known to frequently forage near streetlamps in Italian cities (Tomassini et al. 2014; Russo and Ancillotto 2015; Russo et al. 2019), thereby increasing foraging opportunities. We did not formulate specific predictions for light‐averse species, as they are rare or largely excluded from highly urbanized areas such as Naples. Therefore, our light‐related hypothesis primarily reflects the expected response of the dominant synurbic assemblage, and we predicted higher bat activity in illuminated areas.
2. Urban land use, especially when the urban matrix includes urban natural parks, would provide key foraging and roosting resources. First, it would advantage opportunistic bat species such as pipistrelles, as they often dominate urban bat assemblages (Russo and Ancillotto 2015). Besides, the city's green spaces may act as small‐scale biodiversity hotspots, providing niches for more specialized bat species (e.g., Villarroya‐Villalba et al. 2021). Therefore, we predicted that more urbanized areas and greater extents of natural urban parks would support higher bat activity and species richness.
3. The urban heat island effect (e.g., McGlynn et al. 2019) would buffer temperature variation across seasons, limiting seasonal declines in activity. Thus, we predicted that bat activity would persist across the year, showing weak seasonal patterns.

## 2 Materials and Methods

### 2.1 Study Sites and Land Use Classification

**FIGURE 1:**
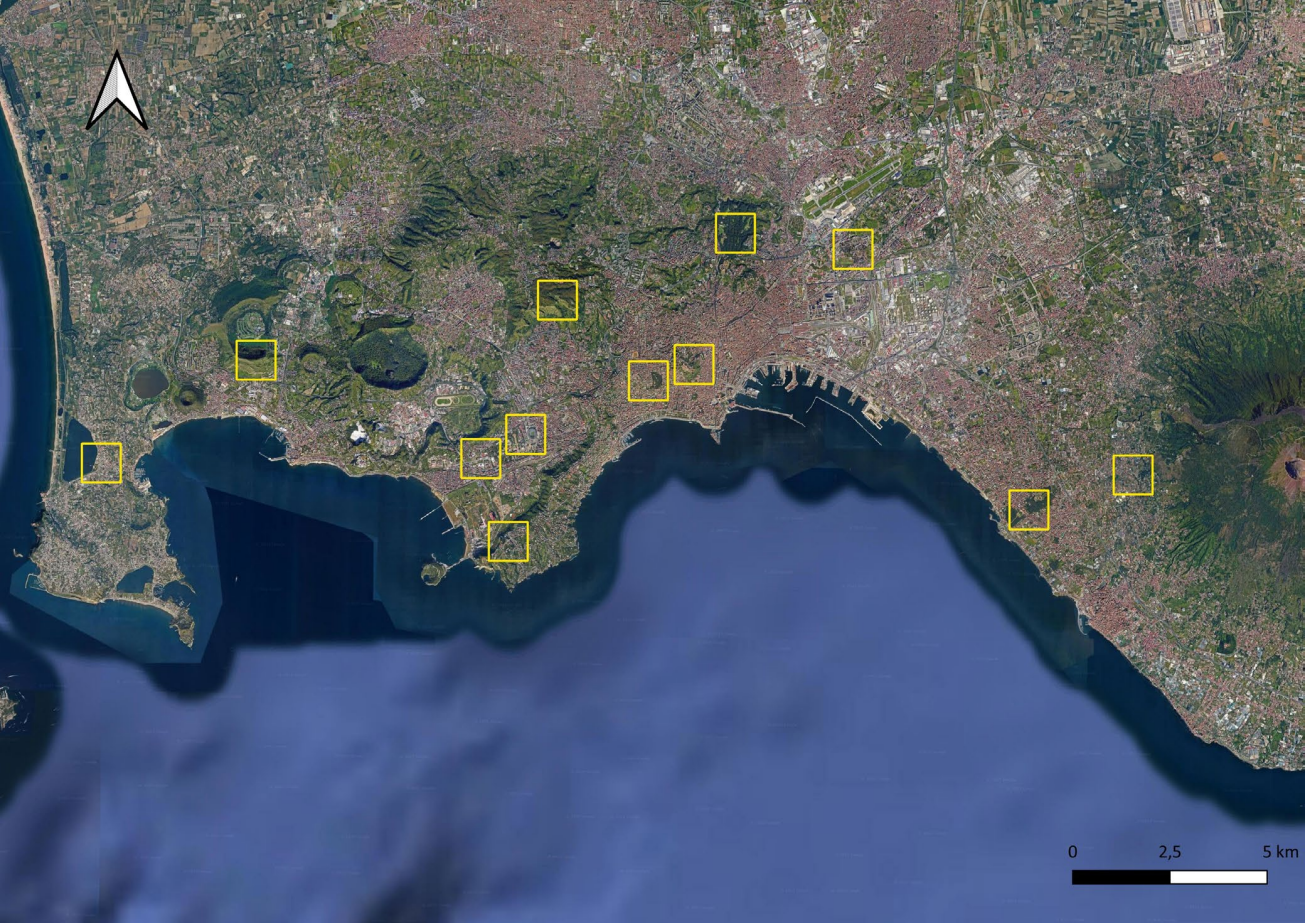
The location of the twelve 1 × 1 km cells where bat richness and activity were sampled in Naples' metropolitan area (Italy).

We set our study in Naples' metropolitan area, southern Italy (40°50′49.20″ N and 14°15′54.00″ E). Naples is the third‐largest Italian city, with over 959,000 inhabitants (Fraissinet et al. 2023; Figure 1). The climate is typically Mediterranean. Naples' metropolitan area lies at the heart of the gulf that shares its name, overlooked by the volcanic massif of Mount Vesuvius. To the east, it is bordered by the Sorrentine Peninsula, ending at Punta Campanella; to the west by the Phlegraean Fields, including Monte di Procida; and to the northwest‐east by the southern section of the Campanian plain, which stretches from Lake Patria to the Nola region. In Naples, urban green spaces and parks represent a relatively small percentage of the city's land area. The main city's area features approximately 50 parks and green spaces, hosting an estimated number of 50,000 trees (Comune di Napoli 2021). Despite these numbers, Naples ranks low among Italian cities, with an estimated average of six trees per 100 inhabitants. This is partly due to the high urbanization of the area, which leaves limited space for green areas. Overall, urban green cover is limited in Naples, with efforts ongoing to balance urbanization and the need for green spaces. The city's tree planting rate slightly exceeds tree removal, but the total green cover remains minimal compared to other cities (Pace et al. 2023). While the city of Naples itself is densely urbanized, the surrounding areas feature patches of arable land and pastures. These areas are often in peri‐urban zones or rural municipalities adjacent to Naples. However, agricultural lands have been experiencing a gradual decline due to urban expansion and reduced maintenance of traditional farming areas.

We followed the shared protocol described by Dondina et al. (2025) to select sampling sites along a gradient of green area size and fragmentation. A 1 × 1 km^2^ reference grid (https://www.eea.europa.eu/data‐and‐maps/data/eea‐reference‐grids‐2) was applied using the appropriate UTM system (WGS 84‐UTM 33N). Green area metrics were derived from the ISPRA 2021 National Land Cover Map (https://groupware.sinanet.isprambiente.it/uso‐copertura‐e‐consumo‐di‐suolo/library/copertura‐del‐suolo/carta‐di‐copertura‐del‐suolo) and processed in FRAGSTATS (McGarigal 1995). Within each grid cell, green area size was computed as the total surface of intersecting green patches and classified into four size categories (A: 0–0.02 km^2^, B: 0.02–0.24 km^2^, C: 0.24–1 km^2^, D: > 1 km^2^). Fragmentation was quantified using the Aggregation Index (AI) within a 1.5 km buffer centered on each cell, and cells were grouped into four fragmentation classes (1: AI < 67; 2: 67–73; 3: 73–81; 4: AI > 81), ranging from highly to minimally fragmented. Combining size and fragmentation classes yielded 16 possible combinations. The specific cells sampled in Naples were not chosen ad hoc but assigned to us by the NBFC project task leaders as part of a standardized national design developed for the National Recovery and Resilience Plan (NRRP—NextGenerationEU). This procedure maximized the representativeness of green area size–fragmentation classes across Italian cities. Details of this gradient‐based sampling framework are provided in the supporting information of Dondina et al. (2025), which also explains how cells were prioritized to ensure comparability across cities. Proximity to other geographical features (e.g., roads, water bodies) was not explicitly used as a selection criterion but may be indirectly reflected in the fragmentation metrics. In Naples, the final set we used for the analysis included 12 cells (Figure 1).

We classified land use composition within each cell using the Corine Land Cover 2019 classification and pooled similar land uses together for subsequent analysis. We used both CORINE Land Cover's Levels 2 and 3. Level 2 allowed for a broader land cover classification, providing a general understanding of the landscape composition. To gain a more detailed and specific insight into habitat use, where needed, we also incorporated Level 3, which offers finer resolution by distinguishing subcategories within each broader land cover class. The final land use classification was: (a) *Urban natural parks*: Forests (3.1); (b) *Green urban spaces*: Artificial, non‐agricultural vegetated areas (1.4); (c) *Urban areas*: Discontinuous urban fabric (1.2) + Continuous urban fabric (1.1); (d) *Mediterranean shrublands (low maquis and garrigue)*: Sclerophyllous vegetation (3.2.3); (e) *Agricultural areas*: Permanent crops (2.2) + Pastures (2.3); (f) *Open rural areas*: Land principally occupied by agriculture, with significant areas of natural vegetation (2.4.3) + Agro‐forestry areas (2.4.4).

We categorized the number of lights within a 150 m radius from the sampling point as “None” = 0, “Low” = 1–2, “High” = 3–5 as a fixed predictor to represent local light pollution levels.

### 2.2 Ultrasonic Recordings and Sound Analysis

We surveyed bat activity from June 2023 to February 2024 using AudioMoth passive ultrasonic recorders, mounted on vertical supports at 1.5–2 m height. We refrained from recording on rainy nights or in strong wind conditions. Devices were programmed to activate 30 min before sunset and deactivate 30 min after sunrise each night. We adopted the following settings: no‐rest period, sampling rate = 384 kHz, and mid gain. Within each cell, we recorded simultaneously at three randomly selected points, ca. 500 m apart. For analytical purposes, we grouped sampling dates into two seasons: “summer–early autumn” (hereafter referred to simply as “summer,” June 7–October 9) and “winter” (February, corresponding to the coldest period in Naples). Each cell was surveyed during four one‐night sessions (two in summer and two in the winter), resulting in 144 survey points. Up to 3 cells were surveyed together on a single night. Sampling nights were intentionally distributed across the entire season rather than conducted consecutively to capture temporal variability in bat activity. Bat echolocation calls in recordings were identified using Kaleidoscope Pro (Wildlife Acoustics Inc.) with a customized species list restricted to bats known to occur in peninsular Italy. Default classification filters were applied, and about 10% of all files, particularly those containing unusual or uncertain responses, were manually vetted to validate species assignments. Due to the low confidence in *Myotis* species identification, all calls from this genus were pooled together for analysis.

### 2.3 Statistical Analysis and Model Fitting

To analyse the relationship between predictor variables and bat activity levels or species richness, GLMMs were employed using the glmmTMB package in R (Brooks et al. 2017). Poisson distributions with a logarithmic link function were considered, as this approach is standard for count data. However, diagnostic tests revealed overdispersion, violating the assumption of equidispersion required for Poisson models. To address this issue, a negative binomial distribution was used for activity data (bat passes), and a Conway‐Maxwell Poisson distribution was applied for species richness data, as both distributions introduce additional parameters to account for overdispersion (Hardin and Hilbe 2007). For activity variables (total passes and those of the most abundant species), a mean‐parametrised negative binomial GLMM was developed, with bat passes as the response variable. For species richness, a mean‐parametrised Conway‐Maxwell Poisson GLMM was developed. The surface area (km^2^) of each of the six land‐cover categories identified within each sampling cell (urban woodland, green urban spaces, urban areas, Mediterranean shrubland, agricultural areas, and open rural areas) was calculated and used as a continuous predictor variable in the models. To ensure linear relationships with the response variables and address scale differences, such six numeric habitat area were log10‐transformed (+1). The code of each sampling site (SiteID) and the date of each sampling (Date) were included as random effects to account for unmeasured spatial and temporal variability and the hierarchical structure inherent to the data. The incorporation of SiteID facilitates the partitioning of variance attributable to site‐level differences, while Date offers a partial capture of nightly environmental variability that remains unmeasured or unused in the models (Bolker et al. 2009). To account for any differences in sampling effort due to variable recording durations among nights, log‐transformed night duration (in hours) was included as an offset term in the models. This approach effectively modeled the bat pass rate per hour, whilst controlling for variation in the night duration for each sampling attempt and obviating the necessity for transformation of the response variables. Interactions between season and land use area variables were tested to determine if their influence varies seasonally and should be included in the final models. Multicollinearity among predictors was assessed using variance inflation factors (VIFs), calculated with the “check collinearity” function from the performance package. Temperature and season were strongly correlated and confounded, as temperature distributions were entirely non‐overlapping between seasons. Additionally, temperature variation was partially nested within site and season effects, offering little independent explanatory power beyond what was captured by the season factor. Thus, to avoid multicollinearity and improve the parsimony of the model, we excluded mean daily temperature as a fixed effect and retained only season (Zuur et al. 2009).

Model selection followed a combination of forward and backward stepwise approaches. All possible interactions (e.g., Season × UrbanAreas) were included in separate models to assess their relevance. Forward selection began with null models, with predictors incrementally added to identify those significantly improving model performance. Concurrently, backward selection was initiated with full models, whereby non‐significant predictors (*p* > 0.05) were removed based on ANOVA type‐III likelihood ratio tests and Akaike information criterion (AIC) values. When AIC differences between nested models were negligible (ΔAIC < 2 and no significant likelihood ratio test), simpler models were favored. The performance of each model was further evaluated using pseudo‐*R* ^2^ values calculated via the Nakagawa method (Lüdecke et al. 2021). Pseudo‐*R* ^2^ values are particularly suitable for complex models such as GLMMs, where traditional *R* ^2^ metrics are undefined or inadequate. Marginal *R* ^2^ values represent variance explained by fixed effects, while conditional *R* ^2^ values include variance explained by both fixed and random effects. The DHARMa package (Hartig and Lohse 2022) was used to conduct diagnostics, including residual plots and statistical tests, confirming the adequacy of model fit. The effects package (Fox and Weisberg 2018) was used to visualize and interpret the effects of the significant predictors for the final models, providing plots that show how the predicted response variable changes across values of one predictor, while holding other variables constant. All analyses were performed in R (version 4.4.1; R Core Team 2024), with key packages including glmmTMB (Brooks et al. 2017), DHARMa (Hartig and Lohse 2022), effects (Fox and Weisberg 2018), and performance (Lüdecke et al. 2021). Only significant predictor variables were visualized in the figures.

## 3 Results

Seven bat species and one species group (*Myotis* sp.) were identified across 36 sampling locations within 12 sites/parks (Appendix S1). The highest total number of bat passes (5695) was recorded at a holm oak woodland during the summer period; while in one winter sampling in an open/rural area, no bat passes were observed. The species with the highest recorded activity was *Hypsugo savii* , with 4123 passes observed at the above‐mentioned holm oak woodland site on a single night (Table 1). However, *Pipistrellus kuhlii* exhibited the highest mean activity across all sites (Table 1). *Pipistrellus pipistrellus* and *Hypsugo savii* demonstrated comparable mean activity levels, while *Tadarida teniotis* recorded the lowest mean number of passes among the most common species (Table 1). In contrast, some species, including *Eptesicus serotinus* , *Plecotus austriacus* , *Rhinolophus ferrumequinum* , and *Myotis* sp., were exceedingly rare. These species were recorded in only a few cells and were excluded from further analysis.

**TABLE 1:** Descriptive statistics for bat activity and species richness recorded in the Naples city area. Mean, standard deviation (SD) and maximum and minimum nightly values/cell are shown.

Variable	Mean	SD	Min	Max
Total number of passes	588.3	991.5	0	5695
Species richness	3.8	1.3	0	8
*Eptesicus serotinus*	0.2	1.1	0	10
*Hypsugo savii*	121.8	409.6	0	4123
*Myotis* sp.	0.6	1.8	0	15
*Pipistrellus kuhlii*	311.6	555.6	0	3218
*Pipistrellus pipistrellus*	122.7	268.7	0	1897
*Plecotus austriacus*	0.1	0.5	0	4
*Rhinolophus ferrumequinum*	1	9.9	0	119
*Tadarida teniotis*	30.3	117.9	0	1102

**FIGURE 2:**
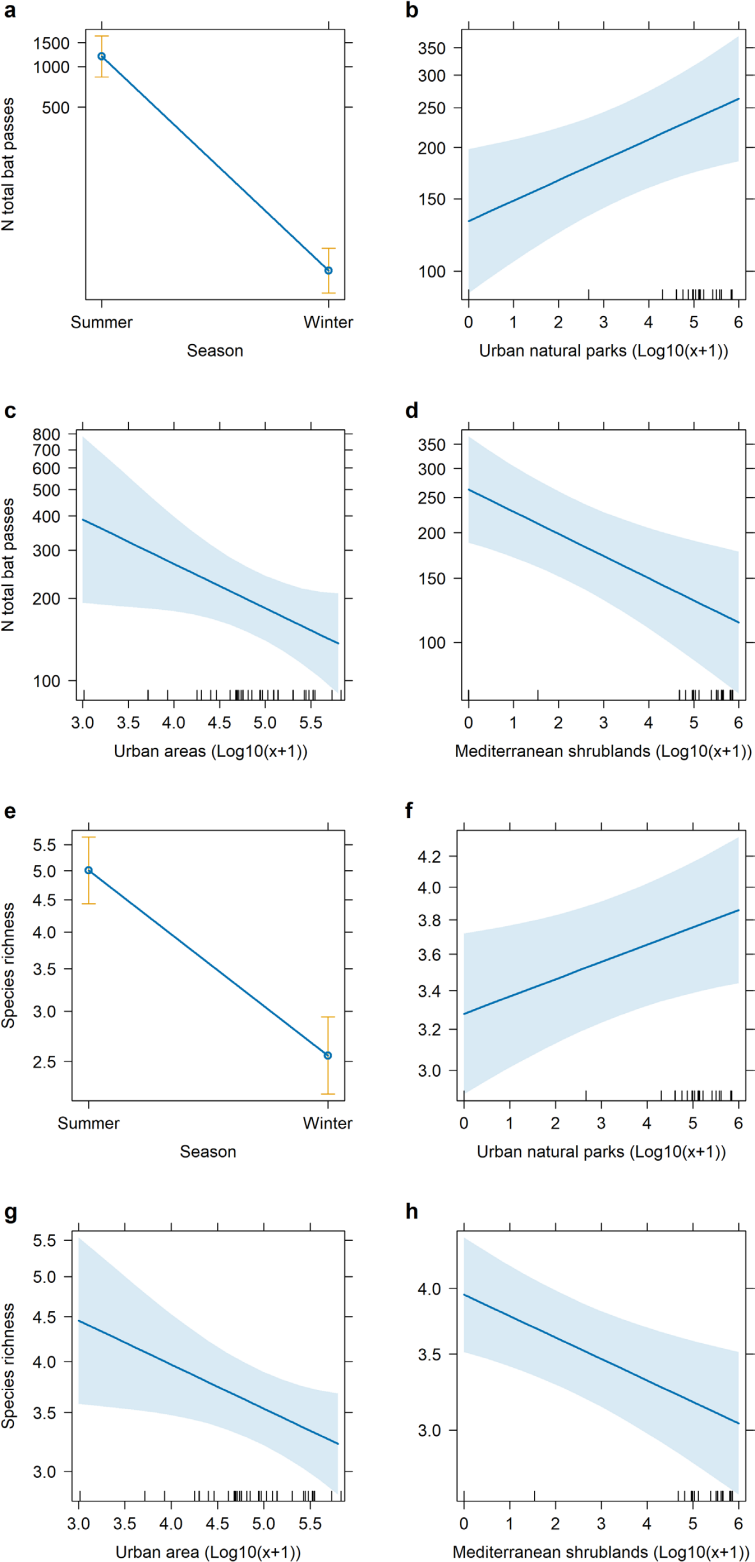
Predicted number of total bat passes and species richness (mean ± SE) in the metropolitan area of Naples, Italy, based on generalized linear mixed models (GLMMs). Total bat activity by (a) Season, (b) extent of Urban natural park areas, (c) extent of Urban areas, and (d) extent of Mediterranean shrubland areas. Species richness by (e) Season, (f) extent of Urban natural park areas, (g) extent of Urban areas, and (h) extent of Mediterranean shrubland areas.

AIC values and pseudo‐*R* ^2^ statistics for the best GLMMs are summarized in Table 2. The best‐fitting model for total bat passes, as determined by significant predictors and low AIC values, included Season, Urban natural parks, Urban areas, and Mediterranean shrublands. Total activity was higher during warmer periods (summer) and positively associated with Urban natural parks, while Urban areas and Mediterranean shrublands had a negative influence (Figure 2). The optimal richness model incorporated Season, Urban natural park areas, Urban areas, and Mediterranean shrublands (Figure 2). As for total activity, species richness was higher during the summer and in Urban natural parks, while greater extents of Urban areas and Mediterranean shrublands negatively affected species richness.

**TABLE 2:** Statistics for the best models of bat activity and species richness in the Naples metropolitan area.

Dependent variable	Predictors	Estimate (*β*)	*p*	AIC	Marginal *R* ^2^	Conditional *R* ^2^
Total Number of Passes	(Intercept)	6.402	< 0.001	1848.362	0.802	0.861
	Season (winter)	−3.656	< 0.001			
	Urban natural parks	0.114	0.011			
	Urban areas	−0.373	0.034			
	Mediterranean shrublands	−0.140	0.003			
Richness	(Intercept)	−0.270	0.334	458.460	0.322	0.443
	Season (winter)	−0.674	< 0.001			
	Urban natural parks	0.027	0.030			
	Urban areas	−0.115	0.027			
	Mediterranean shrubland	−0.043	0.003			
*Hypsugo savii*	(Intercept)	2.130	< 0.001	1307.112	0.640	0.850
	Season (winter)	−3.700	< 0.001			
	Urban natural parks	0.160	0.016			
*Pipistrellus kuhlii*	(Intercept)	4.055	< 0.001	1671.708	0.735	0.735
	Season (winter)	−3.465	< 0.001			
*Pipistrellus pipistrellus*	(Intercept)	2.464	< 0.001	1285.323	0.782	0.845
	Season (winter)	−4.060	< 0.001			
	Open rural areas	0.226	< 0.001			
	Agricultural areas	−0.217	< 0.001			
*Tadarida teniotis*	(Intercept)	0.726	0.072	1032.875	0.516	0.549
	Season (winter)	−1.766	< 0.001			
	Urban natural parks	0.147	0.023			
	Mediterranean shrubland	−0.204	0.002			

**FIGURE 3:**
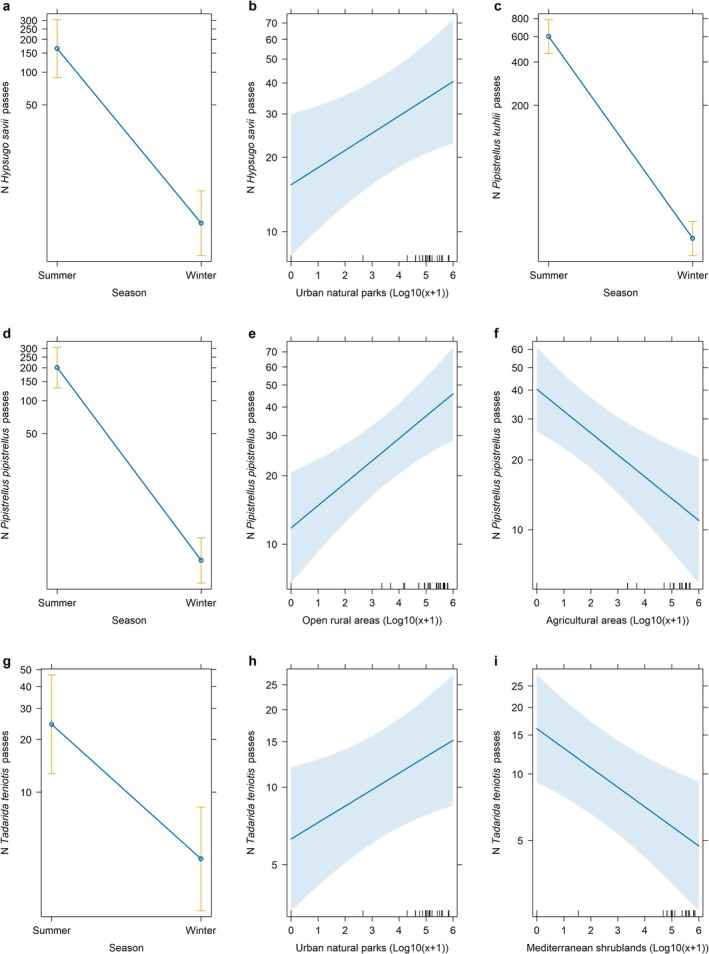
Predicted number of bat passes (mean ± SE) in the metropolitan area of Naples, Italy, based on generalized linear mixed models (GLMMs). *Hypsugo savii* passes by (a) Season, and (b) extent of Urban natural park areas. *Pipistrellus kuhlii* passes by (c) Season. *Pipistrellus pipistrellus* passes by (d) Season, (e) extent of open rural areas, and (f) extent of Agricultural areas. *Tadarida teniotis* passes by (g) Season, (h) extent of Urban natural park areas, and (i) extent of Mediterranean shrublands.

The best‐fitting model for *H. savii* included Season and Urban natural park areas as significant predictors, with higher activity in summer and a positive influence from Urban natural parks (Figure 3). The optimal model for *P. kuhlii* activity identified Season as the sole significant predictor, with significantly higher activity in summer (Figure 3). For *P. pipistrellus* , the optimal model incorporated Season, Open rural areas, and Agricultural areas as predictors, with activity exhibiting a marked increase during summer, a positive influence from Open rural areas, and a negative effect from Agricultural areas (Figure 3). Finally, the best *T. teniotis* model included Season, Urban natural parks, and Mediterranean shrublands (Figure 3). Activity was higher during warmer periods and in Urban natural park areas, while Mediterranean shrublands had a negative influence on bat activity.

## 4 Discussion

Many studies have analyzed bat activity in urban areas in the last decades (e.g., Russo and Jones 2003; Gehrt and Chelsvig 2004; Johnson et al. 2008; Threlfall et al. 2011; Dixon 2012; Li and Wilkins 2014; Straka et al. 2016; Schimpp et al. 2018; Moretto et al. 2019; Li and Kalcounis‐Rueppell 2018; Li et al. 2020; Kohyt et al. 2021; Thomas et al. 2021; Meramo et al. 2025). However, our study provides the first systematic analysis of bat habitat use in a major Mediterranean urban landscape. The bat community featured seven species and one species group (*Myotis* sp.), showing limited species richness. Moreover, the community was dominated by a few synurbic, light‐tolerant species that also occur in other Italian cities, especially *P. kuhlii* , *H. savii* , and *P. pipistrellus* (Russo and Ancillotto 2015; Ancillotto et al. 2024, 2025), while *T. teniotis* was rarer. The remaining species, associated with more natural environments and especially woodland, such as *R* *.* ferrumequinum, *Myotis* sp. and *P. austriacus* , were only occasionally recorded. The rarity of these species likely reflects their ongoing ecological marginalization, a phenomenon increasingly documented among mammals (Britnell et al. 2023) and may anticipate their eventual disappearance as suitable habitats continue to decline (Ancillotto et al. 2025). In the case of *Plecotus* and rhinolophids, their faint or highly directional echolocation calls reduce the likelihood of acoustic detection (e.g., Russo, Ancillotto, and Jones 2018); however, these species are genuinely rare in large Italian cities (D. Russo, pers. obs.). Our findings support the view that urban landscapes function as environmental filters, favoring only those species whose ecological and behavioral traits are compatible with urban conditions (Evans et al. 2018; Jung and Threlfall 2018; Santini et al. 2019).

Contrary to our prediction, ALAN was not an important factor in the analysis and did not feature in the best models, even for streetlamp foragers such as *P. kuhlii* and *H. savii* . Our design did not account for specific light characteristics, such as intensity, spectrum, or lamp type (e.g., LED vs. sodium‐vapor), which are known to influence bat responses (Spoelstra et al. 2017; Voigt, Rehnig, et al. 2018). This limitation may have partially affected our ability to detect species‐specific responses to light. Moreover, the effects of ALAN are difficult to disentangle from the broader influence of urban land cover, which inherently includes higher streetlight density and other correlated factors. In our models, urban areas emerged as a significant negative driver of bat activity and richness, and any potential ALAN effect may be embedded within this more general urbanization signal.

Streetlamps may positively influence the activity of light‐tolerant species such as *P. kuhlii* by concentrating positively phototactic insects such as moths (Tomassini et al. 2014). While some studies have suggested that *H. savii* exploits urban streetlamps for foraging (Vernier 1995; Paunović et al. 2015), this preference was not confirmed by more recent radio‐tracking work (Ancillotto et al. 2018; Kipson et al. 2018). Instead, the species avoids illuminated areas when more natural habitats with ample insect prey are accessible (Kipson et al. 2023). In England, *P. pipistrellus* (the third most frequently recorded species in our study area) showed reduced activity in illuminated compared to dark transects, except in areas with high tree density; in Ireland, its activity appeared insensitive to lighting (Mathews et al. 2015). A further British study also found a negative response of common pipistrelle activity to urbanization (Lintott et al. 2016).

Both total bat activity and species richness were negatively influenced by the extent of urban areas and Mediterranean shrublands, but increased in the presence of urban natural parks. These parks also had a positive effect on *Hypsugo savii* and *Tadarida teniotis* , with the latter species showing reduced activity in Mediterranean shrublands. *Pipistrellus pipistrellus* was positively associated with open rural areas but negatively affected by agricultural areas. The extent of urban land cover within the landscape reduced total bat activity and species richness and did not favor the activity of synurbic species, thus rejecting our second hypothesis. This outcome contrasts with previous studies on *P. kuhlii* in Italian urban areas (Serangeli et al. 2012; Ancillotto et al. 2015) but aligns with the known foraging preferences of *Hypsugo savii* (Ancillotto et al. 2018). Serangeli et al. (2012) focused on seminatural landscapes where urban areas were rural and discontinuous, and Ancillotto et al. (2015) examined roost selection and nursery colony productivity, reporting a positive effect of discontinuous—rather than continuous—urban habitat surrounding colonies. Such benefits may be attributable primarily to more favorable roosting conditions rather than enhanced foraging opportunities in semi‐urbanized landscapes. In contrast, much of Naples's urban fabric is continuous, offering fewer roosting and foraging opportunities and thus differing markedly from the contexts examined by Ancillotto et al. (2015).

The negative relationship between urban land cover and species richness confirms urbanization's negative impact on more sensitive species, particularly narrow‐space foragers reliant on natural habitats. Their local extinction is likely driven by habitat loss associated with urban expansion (Ancillotto et al. 2024, 2025). It is worth noting that we pooled *Myotis* species due to substantial overlap in their echolocation calls, which can lead to misidentification. While using a genus‐level category as a surrogate for species may introduce a potential bias in richness estimates, this is likely negligible in our context, given the low expected diversity of *Myotis* bats in urban environments.

Interestingly, different contexts can yield divergent patterns. For example, in Neotropical dry forests, chronic anthropogenic disturbance was associated with reduced bat activity but increased species richness (Meramo et al. 2022). This discrepancy with our results may reflect differences in disturbance type, taxonomic composition, or regional context. It nevertheless underlines the importance of taxon‐specific responses and the need for community‐level approaches to assess the impacts of human disturbance on bats.

In agreement with our hypothesis, however, within the urban matrix, urban natural parks played an important role in sustaining both bat activity and species richness, while also favoring synurbic species such as *H. savii* and *T. teniotis* . This finding reinforces growing evidence that urban green spaces are crucial because they provide ecological niches otherwise unavailable to urban‐sensitive bat species, while also supporting generalists (e.g., Avila‐Flores and Fenton 2005; Tena et al. 2020; Callas et al. 2024). Outside urbanized areas, open rural landscapes with low‐intensity agriculture and heterogeneous agroforestry mosaics offer structurally diverse edge habitats favored by edge specialists like *P. pipistrellus* (Downs and Racey 2006; Nicholls and Racey 2006). In contrast, intensively managed farmland lacks the structural complexity and linear features essential for this species (Verboom and Huitema 1997) and is often subject to pesticide use, both of which reduce insect prey availability (Russo, Bosso, and Ancillotto 2018; Russo et al. 2024).

The decline in *T. teniotis* activity with increasing coverage of Mediterranean shrubland is more difficult to interpret. As this species typically forages high above the ground (O'Mara et al. 2021), it is only weakly influenced by ground‐level habitat structure. Temporary insect aggregations are likely the main factor shaping this species' foraging patterns (Marques et al. 2004). Mediterranean vegetation has sometimes been associated with increased foraging activity (Russo and Jones 2003; Marques et al. 2004), but in studies focused on natural contexts. Within urbanized landscapes, vegetation types may alter prey availability and foraging dynamics in different ways.

Finally, we hypothesized that urban heat island effects would buffer temperature drops and reduce seasonal differences in activity, allowing bats to remain active year‐round. This hypothesis was not supported; seasonality emerged as the most consistent and influential driver across all models. Bat activity and species richness were significantly lower during winter for all species, including presumed urban exploiters such as *P. kuhlii* and *H. savii* . In temperate urban environments, insect populations exhibit marked seasonal fluctuations in abundance, with minimal activity typically observed during winter months (e.g., Fontanarrosa et al. 2009; Adams et al. 2020), likely influencing bat presence and activity. Our findings indicate that, despite the urban warming effect and the generally mild conditions of the Mediterranean climate, clear seasonal declines in bat activity persist in Naples. However, seasonal variation in bat activity across Mediterranean regions is becoming less pronounced in response to climate change (Mas et al. 2022). Since cities are expected to be among the first environments where these shifts become apparent, consistent monitoring of urban bat populations remains especially important.

## 5 Conclusion

Our study reveals that, even within the relatively mild Mediterranean climate, urbanization in Naples results in bat communities with low species richness and activity, dominated by a few generalist species. More sensitive taxa, particularly those reliant on structurally complex or natural habitats, remain rare or largely absent. These patterns suggest that Mediterranean urban environments, despite climatic advantages, provide limited opportunities for a functionally diverse bat assemblage to persist, consistent with global patterns (Jung and Threlfall 2016).

Enhancing habitat quality and connectivity is crucial to support urban bat diversity and maximize ecosystem services such as insect control. Protecting and restoring green infrastructure, reducing ALAN (Voigt, Azam, et al. 2018), and promoting native vegetation and reduced pesticide use (Tena et al. 2020; Callas et al. 2024) can all contribute. Although cities may buffer temperature drops, our findings show that pronounced seasonal variation in bat activity persists. Yet, with ongoing climate change, urban areas may soon become focal points for detecting early phenological and activity shifts in bats. Long‐term, standardized monitoring will be essential to track these changes and guide effective urban biodiversity management.

## Author Contributions

**Vincenzo Meola:** conceptualization (equal), formal analysis (equal), investigation (lead), methodology (equal), writing – original draft (equal), writing – review and editing (equal). **Ioannis Ekklisiarchos:** formal analysis (lead), methodology (equal), visualization (equal), writing – review and editing (equal). **Luca Cistrone:** methodology (lead), visualization (equal), writing – review and editing (equal). **Antonello Migliozzi:** methodology (lead), visualization (equal), writing – review and editing (equal). **Danilo Russo:** conceptualization (lead), formal analysis (equal), funding acquisition (lead), investigation (lead), methodology (lead), supervision (lead), writing – original draft (lead), writing – review and editing (lead).

## Conflicts of Interest

The authors declare no conflicts of interest.

## Supporting information


**Appendix S1:** Dataset used for the richness and habitat use analysis presented in this article.

## Data Availability

All data supporting the findings of this study are provided as Supporting Information. No additional datasets were generated or analyzed.
